# High-Quality Parasitic Disease Laboratory Services Are a Priority at the CDC

**DOI:** 10.4269/ajtmh.22-0235

**Published:** 2022-04-12

**Authors:** Anne E. Purfield, Jay C. Butler, Kevin P. Cain, Wendi Kuhnert, Atis Muehlenbachs, Monica Parise, Jim Pirkle

**Affiliations:** Centers for Disease Control and Prevention, Atlanta, Georgia

The CDC is unwavering in our commitment to provide the highest quality laboratory diagnostic services for parasitic diseases. We clearly hear, understand, and concur with the concerns expressed in the accompanying editorial and appreciate the challenges the pause in testing for parasitic diseases presents for health-care providers, particularly those treating people at elevated risk for parasitic diseases.

We also recognize the crucial role that our agency plays in ensuring those at risk receive equitable services for infections, including those that are generally known to all Americans as well as neglected diseases that are unfamiliar to most Americans. More broadly, the CDC works to protect the global community from parasitic diseases through three main priorities: reducing parasitic disease-related death, illness, and disability in the United States; reducing the global burden of malaria; and eliminating targeted neglected tropical diseases. Our Parasitic Diseases Laboratory is, in many ways, the foundation of this work and serves as a critical resource and often a laboratory of last resort for challenging diagnoses of unfamiliar pathogens when state and private laboratories do not have the relevant testing capacity. Our laboratory experts develop and improve tools and approaches to detect, prevent, and control disease; provide diagnostic assistance and expertise to public health laboratories; and conduct diagnostic tests for parasitic diseases.

None of this work would be possible without our invaluable partnerships with other public health agencies, academia, and clinicians. We recognize that the medical community and the public rely on our Parasitic Diseases Laboratory for accurate and timely diagnostic services and trust us to maintain first-rate standards.

The CDC’s laboratories must meet and maintain the highest standards of excellence to serve as the nation’s premier health protection agency and to maintain the confidence of our citizens, public health experts, and clinical partners. We are working diligently to implement laboratory improvements to ensure we can meet these expectations and continue offering high-quality diagnostic services. Unfortunately, achievement of these improvements has required temporary suspension of some services.

Our highest priority is to resume high-quality testing operations as soon as possible and to offer the same tests that were available before the pause. We are prioritizing diagnostic tests with the greatest public health impact, particularly those assays that are limited in availability outside the CDC. Our Parasitic Diseases Laboratory resumed serologic testing for Chagas disease and morphologic identification of parasites on February 24, 2022 and is on track for phased resumption of the remaining diagnostic assays. Our careful and thorough approach highlights our commitment to quality and our firm belief that persons at risk of parasitic infections deserve the highest quality laboratory services.

We appreciate the patience, support, and acknowledgment of the critical role of the CDC’s Parasitic Diseases Laboratory from our partners and patients. We are constantly working to offer and improve outstanding services. Updates will be communicated as information becomes available on our Diagnosis of Parasitic Diseases website. We thank our partners for their engagement and share their commitment to equitable approaches to the diagnosis, treatment, and prevention of parasitic infections as we work together toward our common goals.

